# The Effect of Trustor Age and Trustee Age on Trustworthiness Judgments: An Event-Related Potential Study

**DOI:** 10.3389/fnagi.2022.815482

**Published:** 2022-03-09

**Authors:** Zi-wei Chen, Yong-na Li, Ke-xin Wang, Yue Qi, Xun Liu

**Affiliations:** ^1^CAS Key Laboratory of Behavioral Science, Institute of Psychology, Beijing, China; ^2^Department of Management, Society and Communication, Copenhagen Business School, Copenhagen, Denmark; ^3^Sino-Danish College, University of Chinese Academy of Sciences, Beijing, China; ^4^Sino-Danish Centre for Education and Research, Beijing, China; ^5^Department of Psychology, Renmin University of China, Beijing, China; ^6^The Laboratory of the Department of Psychology, Renmin University of China, Beijing, China; ^7^Department of Psychology, University of Chinese Academy of Sciences, Beijing, China

**Keywords:** facial age, decision making, trustworthiness judgments, trust game, facial

## Abstract

Perceived trustworthiness based on facial appearance plays an important role in interpersonal trust and cooperative behavior. Interpersonal trust behaviors involve both trustors and trustees. However, there is no clear conclusion on how the age of the two individuals affects interpersonal trust behaviors. Therefore, this study used the trust game task to explore the differences in trust behaviors between two different age groups in response to faces of different ages and analyzed whether such differences were apparent in the face processing stage. The behavioral results showed that only younger adults invested more money with older partners than younger ones; that is, younger adults trusted older faces more. The event-related potential (ERP) analyses showed that in the early stage of face processing, younger faces elicited more negative N170 than older faces; at the same time, older faces elicited more positive VPP than younger faces, and younger adults had more positive VPP than older adults. In the middle and late stages of face processing, younger faces elicited more negative FRN than older faces in younger adults but not in older adults. In addition, older faces elicited more positive LPP than younger faces in older adults but not in younger adults. The neural analyses suggested that age-related differences in facial trustworthiness judgments might occur in the later stages of face processing. Combining the behavioral and neural results, we found a dissociation between trustworthiness perceptions and trust behaviors in both younger and older adults, which may provide insight into how to prevent older adults from being deceived.

## Introduction

People can judge others in a bottom-up way based on their facial cues (Todorov et al., 2009). Among these judgments, the fundamental salient type is that of facial trustworthiness (Oosterhof and Todorov, 2008). Face-based trustworthiness is defined that individuals make trustworthiness judgments based on the extraction of a person’s facial features (Marzi et al., 2014). Individuals can even make decisions on whether a face is trustworthy within a limited time (Todorov et al., 2008). When interacting with a stranger, people may make trustworthiness judgments based on first impressions in a bottom-up way, such as extracting facial cues (Oosterhof and Todorov, 2008; Marzi et al., 2014), and then decide whether to cooperate with this person (Rusman et al., 2012; Leng et al., 2020b). Individuals can trust others and show their own trustworthiness traits to others. Trust and trustworthiness are closely related and are both vital.

Previous studies have used two types of responses, namely, self-reported and behavioral trust measures, to explore face-based trustworthiness. In tasks involving self-reported trust, participants serving as the trustors are required to judge whether trustees are trustworthy (Dzhelyova et al., 2011; Marzi et al., 2014; Calvo et al., 2018), or to assess the trustworthiness of the trustees on a scale (Yang et al., 2011; Bailey et al., 2015a,b). In behavioral trust tasks, participants play an economic game with their partner, such as the trust game (Chen et al., 2012; Qi et al., 2018; Leng et al., 2020b; Li et al., 2021) or the ultimatum game (Osinsky et al., 2014). In the trust game, a participant serving as the trustor chooses an amount of money to give to the trustee, and the trustee chooses a certain portion of the doubled amount to return to the trustor. In the ultimatum game, the allocation of money between two parties is explored, where the proposer proposes a scheme for the allocation of the money, and the responder can either accept or reject the scheme. Although both paradigms are used to explore interpersonal trust, the trust game is more widely used to explore the effects of facial trustworthiness.

Several studies have shown that people make trustworthiness judgments that spontaneously rely on facial expressions and facial structure cues, such as hair style, eye contact, and the ratio of facial width to height (Todorov et al., 2008; van’t Wout and Sanfey, 2008; Stirrat and Perrett, 2010). Additionally, the gender of faces (Oosterhof and Todorov, 2008; Carragher et al., 2018) and attractiveness (Ma et al., 2016; Kaisler and Leder, 2017; South Palomares and Young, 2017) can affect trust judgments. There has been a growing research focus in the recent years on face-based trustworthiness owing to the ability to alter trustees’ facial stimuli. Age, which is another relevant factor of trustees and trustors, plays a crucial role in trust judgments (Bailey et al., 2015a; Poulin and Haase, 2015; Greiner and Zednik, 2019). A previous study hypothesized that automatic evaluations of trustees affect trustors’ behaviors. Specifically, positive evaluations induce approach tendencies, and negative evaluations induce avoidance tendencies (Chen and Bargh, 1999). For example, faces with positive emotion and high attractiveness tend to be considered to have a high level of trustworthiness (Oosterhof and Todorov, 2008; Ma et al., 2016), and consequently, individuals are more likely to willingly approach a person with a smile and cooperate with this individual.

Similar to emotion and attractiveness, facial age has been shown to be associated with approach or avoidance tendencies based on research examining trust judgments. On the one hand, researchers have found that people tend to evaluate older adults more negatively (Ward, 1988; Ebner, 2008), such as lacking attractiveness (Wernick and Manaster, 1984; Ebner, 2008) and having high negative emotions (Sacco and Hugenberg, 2009; Craig and Lipp, 2017). Meanwhile, trustors judge older adults as less competent (Kite and Johnson, 1988) and not able to make fair decisions; thus, they may reduce their cooperation with older adults in economic games. On the other hand, it is likely that individuals have both negative and positive stereotypes about older adults. Boduroglu et al. (2006) found that individuals in Asian cultures are more respectful of older adults and have more positive first impressions of them than they do of younger adults. Furthermore, studies have suggested that compared with younger adults, older adults are thought to have more prosocial attributes, such as wisdom and warmth (Fiske et al., 2002; Boduroglu et al., 2006). Greiner and Zednik (2019) used the trust game and asked participants from different age groups to serve as trustors and trustees. They found that with increasing age, individuals serving as the trustees showed higher levels of trustworthiness for trustors when playing economic games. In addition to the trustee age, the trustor age is another major impact factor on interpersonal trust judgments (Bailey et al., 2015a,b; Kiiski et al., 2016; Bailey and Leon, 2019). Studies have found that as trustors age, they trust others more (Bailey and Leon, 2019; Greiner and Zednik, 2019), and this impact of age on trust judgments appears in self-reported measures (Li and Fung, 2013; Poulin and Haase, 2015). In terms of economic tasks of trustworthiness, an opposite effect of age (Holm and Nystedt, 2005) or no effect have been found (Sutter and Kocher, 2007; Rieger and Mata, 2013; Telga and Lupianez, 2021). Considering the increased external validity by using face photographs as visual stimuli, it is necessary to further explore whether there is an interaction between trustor and trustee age in behavioral trust measures based on facial appearances.

The proposed explanations regarding the effects of age on trust judgments have mainly focused on the acceptance and processing of positive and negative information by different age groups. As dynamic integration theory points out, with increasing age, older adults tend to allocate fewer cognitive resources toward negative information because they cannot tolerate negative feelings (Labouvie-Vief, 2003). Based on this, older adults are less likely to integrate negative information from faces; thus, they trust faces more than younger adults do. Socioemotional selectivity theory suggests that younger adults focus on knowledge acquisition and expanding their horizons, but older adults are concerned with emotional meaning and satisfaction (Lang and Carstensen, 2002; Carstensen, 2006). The differences in goal priorities based on age result in individuals attending to different cues, which alters the processing of information. Based on the above idea, researchers found that older adults tended to process positive information with respect to negative information, and younger adults preferentially processed negative information (Reed et al., 2014). Furthermore, trust judgments based on facial features could originate from memories of past interpersonal interaction experiences (Suzuki and Suga, 2010). Although older adults make decisions relying on growing affective knowledge (Peters et al., 2007), they may still be less influenced by facial cues when making trust judgments. As Bailey and Leon (2019) pointed out, older adults might extract extensive experience from their lifetimes, which allows them to realize the lack of reliability of facial features for trust decisions and makes them less likely to process primary facial features such as facial emotion and facial age. However, if older adults fail to retrieve memories from their extensive personal experiences to restrain their impluses to make face-based trustworthiness judgments, they might then tend to focus on primary facial features (Samanez-Larkin and Knutson, 2015; Suzuki, 2016). There are still no consistent conclusions regarding the influences of trustor and trustee age on trust judgments.

Although much research has focused on the effect of trustor and trustee age in face-based trust judgments, some research has not found an impact of trustor age on behavioral trust measures (Sutter and Kocher, 2007; Rieger and Mata, 2013; Telga and Lupianez, 2021). Using the electroencephalogram (EEG) technique, researchers can further explore the cognitive mechanisms and temporal dynamics of trustworthiness perceptions in different age groups to determine whether individuals extract and process facial age cues. Recent EEG studies on facial trustworthiness have shown that some early components of face processing also change when individuals are making trust judgments. First, as a classic early negative component observed in face processing, N170 reflects the structural encoding of faces in the occipitotemporal region (Bentin et al., 1996; Bentin and Carmel, 2002; Rossion and Jacques, 2012). Tortosa et al. (2013) found that the N170 amplitude increased when seeing a black face compared to a white face during the trust game. The researchers suggested that the enhancement of N170 reflected an increase in the need to extract facial features from other ethnic groups during trust judgments. Studies have also suggested that the enhancement of N170 represented greater focus on facial structures and more complex encoding of faces (Marzi et al., 2014). Dzhelyova et al. (2011) have suggested that when asking participants to make trust judgments in relation to different genders of faces, the N170 amplitude was larger for trustworthy female faces than for untrustworthy female faces, but was larger for untrustworthy male faces than for trustworthy male faces. Thus, researchers have suggested that the N170 amplitudes are larger only when the faces match an existing bias: female faces are more trustworthy and male faces are less trustworthy. In addition, there was a difference between the left and right hemispheres in the magnitude of the N170 component during trust judgments, with the N170 amplitude being larger in the right hemisphere (Bentin et al., 1996). This may be due to the corresponding activation in the right amygdala and superior temporal sulcus during automatic perceptions of trustworthiness (Winston et al., 2002; Engell et al., 2007). At the same time, the vertex positive potential (VPP) is a positive component that is activated in the central region at a time similar to that of the N170 component (150–200 ms), and it also reflects automatic perceptions of facial structure. Some studies have found that when asking participants to make gender judgments about younger faces and older faces, the main effect of facial age was found in the VPP component: older faces elicited more positive VPP, even though the effect was not found for the N170 component (Ebner et al., 2010). Although many studies have suggested that the N170 and VPP components are derived from occipitotemporal lobe activation (Ebner et al., 2010; Luo et al., 2010; Tortosa et al., 2013), some researchers have proposed that the N170 and VPP components represent two different stages of face processing (Joyce and Rossion, 2005). Therefore, more comprehensive results can be obtained by separately analyzing the two components.

In addition to the early face processing components, the middle and late components, such as the FRN (feedback-related negativity) and LPP (late positive potential), are also involved in trust judgments. The FRN, or FN, is a negative wave between 200 and 350 ms that is found in the prefrontal cortex and mainly reflects processing in the feedback stages of economic tasks (Chen et al., 2012; Leng et al., 2020b). Studies have found that the FRN reflects the difference between the expected results and the actual results. The larger the FRN amplitude is, the greater the difference between the expected results and the actual results (Gehring and Willoughby, 2002). In addition, some studies have analyzed the FRN amplitude in the face presentation stage of economic tasks, and they found that the FRN amplitude was larger for those who were unwilling to invest (Li et al., 2017). At the same time, another study found that in the face presentation stage of the ultimatum task, the FRN amplitude was larger when seeing the faces of those presenting unfair assignments (Osinsky et al., 2014). Osinsky et al. (2014) suggested that the FRN originated from the medial frontal cortex and represents the evaluation of the social reputations of faces. Based on this notion, the activation of the FRN component during the face presentation stage may represent a positive or negative evaluation of faces. Regarding the late stage of face processing, related studies have tended to focus on the LPP components. The LPP reflects the processing of the cerebral cortex in working memory representations, decision-making, and reaction (Schupp et al., 2006). In addition, some studies have shown that the LPP is enhanced with increasing selective attention (Schupp et al., 2010; Chen et al., 2012). Yang et al. (2011) found that untrustworthy faces elicited the LPP more positively than trustworthy faces, and the amplitude of the LPP was highly correlated with the trustworthiness of the faces. Regarding the behavioral aspects, individuals tended to stay away from those with untrustworthy faces while paying more attention on them due to vigilance (Yang et al., 2011; Marzi et al., 2014).

The aging phenomenon has gradually become a global challenge in the twenty-first century (Li and Fung, 2013), and older adults rely more on others due to physical and cognitive declines (Bookwala, 2011). Therefore, it is necessary to systematically explore the influence of trustor and trustee age on interpersonal trust behaviors. Individuals make trust judgments based on facial features, but at the same time, such facial trust judgments can also make individuals more vulnerable to deception (Suzuki and Suga, 2010). The purpose of this study was to explore whether there were differences in the behavioral and neural aspects of trust judgments based on facial age between participants in two different age groups. Based on the theory of Bailey and Leon (2019), we expected that there would be an impact of facial age on trustworthiness only in younger adults. Using event-related potential (ERP) analyses, we could further explore the temporal dynamics of trustworthiness perceptions. If younger and older adults automatically extracted facial age cues during the facial processes before they make trustworthiness judgments, we could find the differences in early components, such as the N170 and VPP, elicited by younger and older faces. According to previous studies (Dzhelyova et al., 2011; Tortosa et al., 2013), we expected that the N170 and VPP amplitudes elicited by younger faces would be larger than those elicited by older faces in both younger and older adults. Considering that the FRN amplitude can be used as an indicator of social evaluation (Pegna et al., 2019), and that was expected to be larger when seeing a face with negative attributions (Osinsky et al., 2014), it was likely that there would be an impact of facial age on FRN amplitude only in younger adults. Furthermore, a previous study suggested that older adults extract extensive experience from their lifetimes, which makes them less likely to process primary facial features (Bailey and Leon, 2019). Thus, we expected that the LPP amplitudes elicited by older faces would be larger than those elicited by younger faces in older adults. The findings would also provide empirical evidence for reducing credulity based on facial cues.

## Materials and Methods

### Participants

A total of 54 participants including 29 younger adults (13 women; *M*_age_ = 21.81 years, *SD* = 3.09 years; range 17–30 years) and 25 older adults (15 women; *M*_age_ = 62.08 years, *SD* = 4.20 years; range 55–73 years) participated in this study. All participants were right-handed and reported to have normal color vision, and all of them gave informed consent. We estimated the required size as follows: The main effects of age in trust game by Bailey et al. (2015a) had the effect size of η*_*p*_*^2^ = 0.13, we need at least 38 participants (19 younger adults and 19 older adults) to obtain a desired statistical power of 0.90 for main effects, with an alpha value of 0.05. This study was approved by the internal review board of the Institute of Psychology, Chinese Academy of Sciences.

### Stimuli

When exploring the trustworthiness based on faces, some studies used computer-generated faces (Ma et al., 2016; Bailey and Leon, 2019), but there were more studies using photographs of real faces (Bailey et al., 2015a; Craig and Lipp, 2017; Kaisler and Leder, 2017; Qi et al., 2018). Given the lack of fidelity of computer-generated faces, a total of 96 frontal photographs of Chinese faces were selected, which include 48 older and 48 younger faces with different emotions (happy/neutral/sad), and half of the face photographs were men. Both younger and older faces consisted of 16 happy, 16 emotional neutral, and 16 sad faces. Younger faces are between 17 and 23 years old, whereas older faces are between 60 and 70 years old. Younger faces were selected from Chinese Affective Picture System (Bai et al., 2005), and older faces were used from a previous study (Li et al., 2021). All the face photographs were converted to grayscale and placed on a black background with a size of 260 × 300 pixels according to the direction of the CAPS.

Considering that emotion, expressions and attractiveness might influence trustworthiness judgments (Oosterhof and Todorov, 2008; Ma et al., 2016; Li et al., 2021). In a pilot study, 56 participants (29 women; *M*_age_ = 43.71 years, *SD* = 20.90 years; range 17–73 years) were recruited to rate the emotional valence of younger and older faces which were randomly presented with a 9-level Likert scale, with higher scores indicating higher positive valence of a face. The main effect of facial age was non-significant [*F*(1, 96) = 2.18, *p* = 0.143, η*_*p*_*^2^ = 0.023], which means that there was no significant difference between the emotional valence of younger faces (4.77 ± 1.25) and older faces (5.21 ± 1.62). Another 45 participants (29 women; *M*_age_ = 49.29 years, *SD* = 19.50 years; range 19–73 years) were recruited to rate the attractiveness with a 7-level Likert scale, with higher scores indicating higher attractiveness of a face. The main effect of facial age was non-significant [*F*(1, 96) = 1.45, *p* = 0.231, η*_*p*_*^2^ = 0.015], which means that there was no significant difference between the attractiveness of younger faces (3.77 ± 0.60) and the attractiveness of older faces (3.60 ± 0.72).

### Procedure and Design

**FIGURE 1:**
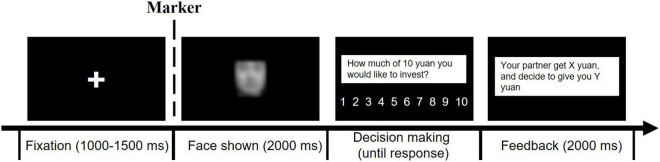
The procedure of single trial.

The stimuli presentation and data collection were controlled by E-Prime 2.0. As shown in Figure 1, the participants were told to play trust games with different people online. In each trial, they would have 10 yuan to invest on their partner in the trial. In each trial, a fixation point “+” was first displayed in the center of the screen for 1,000–1,500 ms, and then, a face photograph was displayed in the center of the screen for 2,000 ms. After the face disappeared, the participants were asked to allocate an amount of money (from 1 to 10 yuan) to that person based on their first impression. The participants were told that their partner would receive quadruple the amount of money invested by the participants and distribute it fairly (5:5) or unfairly (3:1 or the partner could keep all the money). The specific proportions were not provided to the participants. The trial numbers of each combination of facial age (younger faces vs. older faces) and outcomes (gain vs. loss) were identical. After making a decision, the participants would see the distribution results at the end of each trial. Before the formal experiment, the participants were encouraged to use any strategy that they wanted to maximize their number of points. The participants were informed that the monetary bonus would be determined by the actual results of a randomly selected trial. Each face was presented two times (followed by fair feedback in one trial and unfair feedback in another trial), and a practice block with eight trials was completed before the formal experiment to familiarize the participants with the task. The experiment contained four blocks with a total of 192 trials. In each block, the participants played with either younger or older partners. The block sequence was counterbalanced between the participants. At the end of the experiment, one trial was selected randomly, and the actual result of the trial was the bonus money that the participant would receive. Thus, after the experiment, the participants received a fixed monetary compensation for their time and an additional bonus depending on the actual results of the randomly selected trial.

### Behavioral Analysis

In this study, we focused on the effect of trustor and trustee age on trustworthiness judgments. We conducted a 2 (facial age: younger faces vs. older faces)**×** 2 (participants’ age: younger adults vs. older adults) mixed-design measures ANOVA for the mean amount of money invested with partners in each condition. The data of four older adults were excluded from the behavioral and ERP analyses due to either the incompletion of the task or too many artifacts in ERPs. The final sample included 29 younger adults and 21 older adults.

### Electroencephalogram Recording

Electroencephalogram signals were recorded from 64 scalp sites according to the International 10–20 system with Ag/AgCl electrodes and AC Amplifier (Synamps, Neuroscan Inc.). The vertical and horizontal electrooculogram (VEOG and HEOG) sites were used to monitor the horizontal eye movements and eyeblinks, and the electrode impedance was kept below 5 kΩ during recoding. EEG signals were acquired by referenced to the left mastoid online and then were recomputed offline against to the right mastoid.

### Event-Related Potential Processing

ERP analysis was conducted using EEGLAB version 12.02.6 for MATLAB R2013b. The data were filtered with a bandpass of 1–30 Hz and digitized with a sampling rate of 500 Hz. EEG was epoched from 200 ms before and 800 ms after the stimulus onset. ERPs were then baseline corrected using the 200 ms prestimulus period. The epochs with blinks, eye movements, or artifacts exceeding ± 75 μV were rejected, and then, the ERPs from two groups were averaged separately for each of four conditions: younger adults to younger faces and older faces, older adults to younger faces and older faces.

During the stage of face presentation, N170, VPP, FRN, and LPP components were of interest. The N170 component was processed at the occipitotemporal sites in the right hemisphere (PO8) and left hemisphere (PO7) during the time window of 150–180 ms after the stimulus onset. The VPP component was processed at the fronto-central site of Cz during the time window of 155–195 ms in younger adults and 165–215 ms in older adults after the stimulus onset. The FRN component was processed at the fronto-central site of FCz during the time window of 240–280 ms in younger adults and 300–340 ms in older adults after the stimulus onset. The LPP component was processed at the electrode of Pz during the time window of 450–600 ms after the stimulus onset. We used a 2 (facial age: younger faces vs. older faces) × 2 (participants’ age: younger adults vs. older adults) repeated measures ANOVA for mean amplitudes of the interested components (e.g., N170, VPP, FRN, and LPP).

## Results

### Behavioral Data Analyses

**FIGURE 2:**
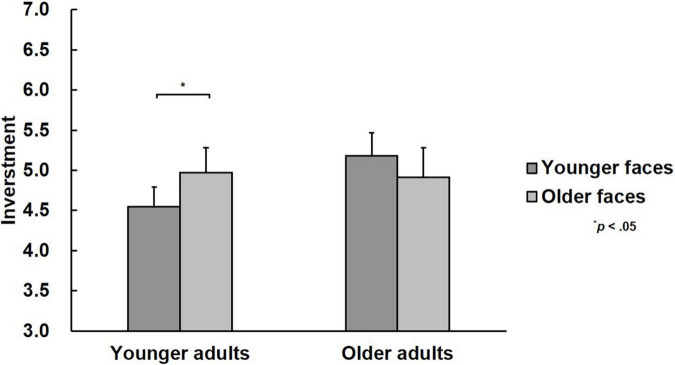
Mean investments in different types of faces.

A 2 (facial age: younger faces vs. older faces)**×** 2 (participants’ age: younger adults vs. older adults) mixed-design ANOVAs revealed an non-significant main effect of facial age [*F*(1, 48) = 0.39, *p* = 0.538, η*_*p*_*^2^ = 0.01] and participants’ age [*F*(1, 48) = 0.49, *p* = 0.487, η*_*p*_*^2^ = 0.01]. But there was a significant interaction between facial age and participants’ age, [*F*(1, 48) = 6.67, *p* = 0.013, η*_*p*_*^2^ = 0.12]. A simple effect analysis showed that more money was invested in older faces (4.97 ± 1.22) than younger faces [4.54 ± 1.43; *F*(1, 48) = 6.10, *p* = 0.017, η*_*p*_*^2^ = 0.11] for younger adults, but no significant difference was found for older adults [*M*_younger faces_ = 5.18 ± 1.69; *M*_older faces_ = 4.91 ± 1.64; *F*(1, 48) = 1.66, *p* = 0.204, η*_*p*_*^2^ = 0.03], as shown in Figure 2.

### Electroencephalogram Data Analyses

#### N170

**FIGURE 3:**
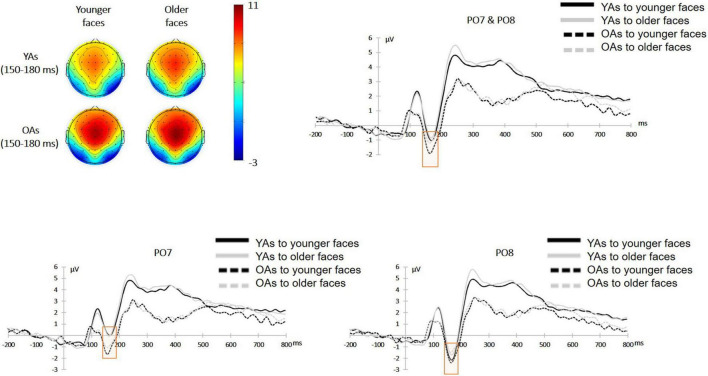
Face-locked ERPs from occipitotemporal electrodes (PO7 and PO8) comparing the N170 component in eight conditions over facial age (younger faces vs. older faces) and participants’ age (younger adults vs. older adults) and electrode (PO7 vs. PO8). Topographical maps showing scalp distribution of the N170 amplitudes in the younger faces (left) and older faces (right) between younger adults (upper) and older adults (lower).

Given the difference of N170 between left and right hemispheres (Bentin et al., 1996; Tortosa et al., 2013), a 2 (facial age: younger faces vs. older faces)**×** 2 (participants’ age: younger adults vs. older adults) **×** 2 (hemisphere: left vs. right) mixed-design ANOVA on the mean amplitude of N170 amplitude revealed a main effect of facial age [*F*(1, 48) = 5.67, *p* = 0.021, η*_*p*_*^2^ = 0.11]. The effect of participants’ age was non-significant [*F*(1, 48) = 0.36, *p* = 0.551, η*_*p*_*^2^ = 0.01]. The effect of hemisphere was significant [*F*(1, 48) = 6.90, *p* = 0.012, η*_*p*_*^2^ = 0.13], and the *post hoc* test indicated that a larger amplitude of N170 in the right hemisphere (–1.61 ± 4.04 μV) than the left one (–0.34 ± 4.27 μV). There was no significant interaction between facial age and participants’ age [*F*(1, 48) = 1.18, *p* = 0.283, η*_*p*_*^2^ = 0.024] or between participants’ age and hemisphere [*F*(1, 48) = 1.81, *p* = 0.185, η*_*p*_*^2^ = 0.04]. However, the interaction between facial age and hemisphere was significant [*F*(1, 48) = 5.36, *p* = 0.025, η*_*p*_*^2^ = 0.10]. The three-way interaction was non-significant [*F*(1, 48) = 0.04, *p* = 0.847, η*_*p*_*^2^ < 0.01]. Figure 3 shows the mean amplitude for each condition.

Considering the interaction between facial age and hemisphere, a simple effect analysis revealed the right hemisphere advantage for both the younger [*M*_left_ = –0.43 ± 4.20 μV; *M*_right_ = –1.92 ± 4.13 μV, respectively, *F*(1, 48) = 8.69, *p* = 0.005, η*_*p*_*^2^ = 0.15] and older faces [*M*_left_ = –0.25 ± 4.37 μV; *M*_right_ = –1.29 ± 3.96 μV, respectively, *F*(1, 48) = 4.67, *p* = 0.036, η*_*p*_*^2^ = 0.09]. Another simple effect analysis showed a significant effect of facial age (i.e., a larger N170 elicited by younger faces than by older faces) only in the right hemisphere [*M*_younger faces_ = –0.43 ± 4.20 μV; *M*_older faces_ = –0.25 ± 4.37 μV, respectively; *F*(1, 48) = 12.45, *p* < 0.001, η*_*p*_*^2^ = 0.21] but not in the left one [*M*_younger faces_ = –1.92 ± 4.13 μV; *M*_older faces_ = –1.29 ± 3.96 μV, respectively, *F*(1, 48) = 0.90, *p* = 0.348, η*_*p*_*^2^ = 0.09].

#### Vertex Positive Potential

**FIGURE 4:**
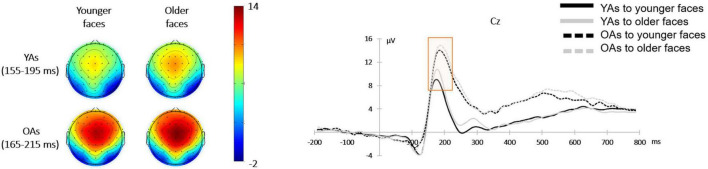
Face-locked ERPs from Cz electrode comparing the VPP component in four conditions over facial age (younger faces vs. older faces) and participants’ age (younger adults vs. older adults). Topographical maps showing scalp distribution of the VPP amplitudes in the younger faces (left) and older faces (right) between younger adults (upper) and older adults (lower).

A 2 (facial age: younger faces vs. older faces)**×** 2 (participants’ age: Younger adults vs. older adults) mixed-design ANOVA on the mean amplitude of VPP revealed a main effect of facial age [*F*(1, 48) = 10.25, *p* = 0.002, η*_*p*_*^2^ = 0.18], and the *post hoc* test indicated that older faces elicited larger VPP amplitude (11.18 ± 6.23 μV) relative to younger faces (10.11 ± 6.05 μV). The effect of participants’ age was also significant [*F*(1, 48) = 8.00, *p* = 0.007, η*_*p*_*^2^ = 0.14], and the *post-hoc* test indicated that older adults activated larger VPP amplitude (13.30 ± 2.45 μV) than younger adults (8.72 ± 1.63 μV). Additionally, there was no significant interaction between facial age and participants’ age [*F*(1, 48) = 0.32, *p* = 0.573, η*_*p*_*^2^ = 0.01]. Figure 4 shows the mean amplitude for each condition.

#### Feedback-Related Negativity

**FIGURE 5:**
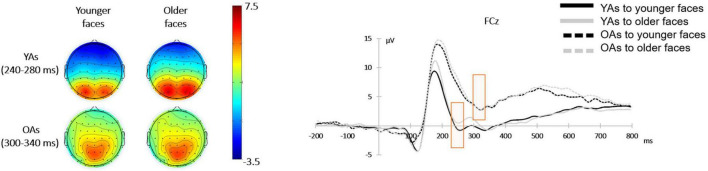
Face-locked ERPs from FCz electrode comparing the FRN component in four conditions over facial age (younger faces vs. older faces) and participants’ age (younger adults vs. older adults). Topographical maps showing scalp distribution of the FRN amplitudes in the younger faces (left) and older faces (right) between younger adults (upper) and older adults (lower).

A 2 (facial age: younger faces vs. older faces)**×** 2 (participants’ age: younger adults vs. older adults) mixed-design ANOVA on the mean amplitude of FRN revealed a main effect of facial age [*F*(1, 48) = 5.76, *p* = 0.020, η*_*p*_*^2^ = 0.11], and the *post hoc* test indicated that younger faces elicited larger FRN amplitude (0.96 ± 4.51 μV) relative to older faces (1.75 ± 4.10 μV). The effect of participants’ age was also significant [*F*(1, 48) = 6.84, *p* = 0.012, η*_*p*_*^2^ = 0.13], and the *post hoc* test indicated that older adults activated larger FRN amplitude (0.11 ± 3.37 μV) than older adults (3.07 ± 4.88 μV). Additionally, there was a significant interaction between facial age and participants’ age [*F*(1, 48) = 4.54, *p* = 0.038, η*_*p*_*^2^ = 0.09]. Figure 5 shows the mean amplitude for each condition.

Considering the interaction between facial age and participants’ age, a simple effect analysis revealed that when showing the younger faces, younger adults activated larger FRN amplitude (–0.54 ± 3.41 μV) than older adults [3.03 ± 5.08 μV; *F*(1, 48) = 8.89, *p* = 0.004, η*_*p*_*^2^ = 0.16]. Similarly, when showing the older faces, younger adults activated larger FRN amplitude (0.76 ± 3.25 μV) than older adults [3.11 ± 4.80 μV; *F*(1, 48) = 4.26, *p* = 0.044, η*_*p*_*^2^ = 0.08]. Additionally, in younger adults, younger faces elicited larger FRN amplitude (–0.54 ± 3.41 μV) than older faces [0.76 ± 4.10 μV; *F*(1, 48) = 12.22, *p* = 0.001, η*_*p*_*^2^ = 0.20]. But in older adults, the FRN amplitudes elicited by younger faces (3.03 ± 5.08 μV) and older faces [3.11 ± 4.80 μV; *F*(1, 48) = 0.03, *p* = 0.861, η*_*p*_*^2^ < 0.01] were not significantly different.

#### Late Positive Potential

**FIGURE 6:**
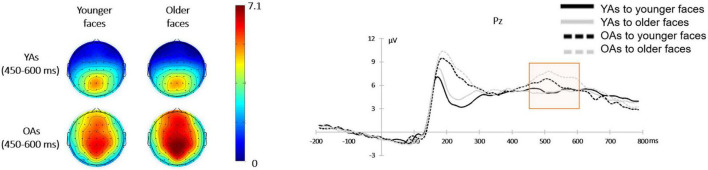
Face-locked ERPs from Pz electrode comparing the LPP component in four conditions over facial age (younger faces vs. older faces) and participants’ age (younger adults vs. older adults). Topographical maps showing scalp distribution of the LPP amplitudes in the younger faces (left) and older faces (right) between younger adults (upper) and older adults (lower).

A 2 (facial age: younger faces vs. older faces)**×** 2 (participants’ age: younger adults vs. older adults) mixed-design ANOVA on the mean amplitude of LPP revealed that no main effect of facial age [*F*(1, 48) = 2.91, *p* = 0.094, η*_*p*_*^2^ = 0.06]. In addition, the effect of participants’ age was also non-significant [*F*(1, 48) = 1.79, *p* = 0.187, η*_*p*_*^2^ = 0.04]. However, there was a significant interaction between facial age and participants’ age [*F*(1, 48) = 6.26, *p* = 0.016, η*_*p*_*^2^ = 0.12]. Figure 6 shows the mean amplitude for each condition.

Considering the interaction between facial age and participants’ age, a simple effect analysis revealed that when showing the younger faces, LPP amplitude activated by younger adults (5.28 ± 3.35 μV) and older adults [6.07 ± 4.25 μV; *F*(1, 48) = 0.54, *p* = 0.466, η*_*p*_*^2^ = 0.01] was not significantly different (*p* = 0.466). Similarly, when showing the younger faces, LPP amplitude activated by younger adults (5.09 ± 3.03 μV) and older adults [7.07 ± 4.35 μV; *F*(1, 48) = 3.57, *p* = 0.065, η*_*p*_*^2^ = 0.07] was not significantly different (*p* = 0.065). Additionally, in younger adults, the LPP amplitudes elicited by younger faces (5.28 ± 3.35 μV) and older faces [5.09 ± 3.03 μV; *F*(1, 48) = 0.38, *p* = 0.543, η*_*p*_*^2^ = 0.01] were not significantly different. But in older adults, older faces elicited larger LPP amplitude (7.07 ± 4.35 μV) than younger faces [6.07 ± 4.25 μV; *F*(1, 48) = 7.64, *p* = 0.008, η*_*p*_*^2^ = 0.14].

## Discussion

We aimed to explore differences in trust judgments based on younger and older faces for participants in different age groups using the trust game. Meanwhile, we further explored whether there were differences during the face presentation stage before the trustworthiness judgments were made. The results showed that younger adults invested more money for older faces than younger faces, but there was no significant difference in the amount of money given to younger and older faces by older adults. When analyzing the ERPs during the face presentation stage, it was found that younger faces elicited larger N170 amplitudes than older faces. At the same time, older faces elicited larger VPP amplitudes than younger faces, and younger adults had more positive VPP components activated than older adults. Furthermore, for younger adults, younger faces elicited larger FRN amplitudes than older faces, whereas for older adults, there was no significant difference in the FRN amplitudes between the two kinds of faces; moreover, younger adults had more negative FRN components activated than older adults. Regarding later face processing, older faces elicited larger LPP amplitudes than younger faces only for older adults.

Regarding behavioral performance, younger adults invested more money in older faces than younger faces, whereas the investments of older adults did not differ between younger and older faces. On the one hand, there was a facial age effect on the investments made by younger adults, which was consistent with the previous findings that facial age affects trust judgments, that is, older faces are more trustworthy than younger faces (Fiske et al., 2002; Slessor et al., 2014). However, some studies did not find an interaction between trustee and trustor age. For instance, Telga and Lupianez (2021) used the trust game and found that there was no impact of partner or participant age. They analyzed the cooperation rates (i.e., whether to invest) instead of the exact investment, which might have caused the difference in results. Although there are some negative stereotypes about older adults, such as lack of conscientiousness (Kite and Johnson, 1988) and attractiveness (Ebner, 2008), prosocial impressions such as intimate and warm impression have been associated with older adults (Fiske et al., 2002; Slessor et al., 2014). In this study, younger adults invested more money for older faces, which indicates that it is likely that the impression of older adults might be more positive and prosocial for these younger trustors. At the same time, studies on facial trustworthiness also found that there were crosscultural differences in trust judgments (Jones et al., 2021). All the participants recruited in this study were Asian, and individuals in Asian cultures have more respect for older adults based on traditional etiquette (Boduroglu et al., 2006), so the negative stereotype associated with older adults would likely be negligible. On the other hand, there was no difference in the amount of money that older adults allocated for younger and older faces, which indicated that older adults did not have stereotypes based on facial age. In addition, Bailey and Leon (2019) also pointed out that due to increasing personal experience, older adults may inhibit themselves from making trust judgments using the primary indicators. Based on this, older adults may be reluctant to make trust judgments automatically and quickly based on facial age. Regarding facial age, there was no difference in the amount of money invested by younger and older adults, which was inconsistent with previous studies showing that individuals become more trusting with age (Bailey and Leon, 2019; Greiner and Zednik, 2019). In contrast, other studies have found that trust judgments are not affected by an individual’s age (Holm and Nystedt, 2005; Sutter and Kocher, 2007; Rieger and Mata, 2013). Bailey and Leon (2019) also noted that the effect of an individual’s age on trust judgments could be found in self-reported task performances rather than in measures of behavioral trust. They suggested that to encourage others to eliminate the negative stereotypes associated with older adults, older adults should present themselves to others in a manner that shows how trustworthy they are. In conclusion, the behavioral results confirmed that younger adults were influenced by facial age when making face-based trust judgments, whereas older adults were not.

Based on the behavioral results, we further analyzed the ERPs in the face presentation stage before the participants made trust judgments and explored whether younger adults or older adults extracted facial age cues to make trust judgments. First, younger faces elicited larger N170 amplitudes than older faces. The N170 component reflects the occipitotemporal region’s perception and encoding of the overall structure of the face (Bentin and Carmel, 2002). The increased amplitude of this component reflects the increased demand for the extraction of facial structure, accompanied by more complex encoding of faces (Tortosa et al., 2013; Marzi et al., 2014). Consistent with previous studies (Bentin et al., 1996), the right hemisphere activated a larger N170 component than the left hemisphere. Moreover, the activation of N170 in the right hemisphere was affected by facial age, which further indicated that both younger and older adults could extract facial age cues in the early stage of face processing for subsequent trust judgments. Meanwhile, it was found that larger amplitudes of N170 were elicited by younger faces than by older faces, which represented an increased encoding demand for younger faces. Second, at the same time, older faces elicited larger VPP amplitudes than younger faces, and younger adults had more positive VPP components activated than older adults. The results indicated that younger adults used more cognitive resources when encoding faces during early face processing, which is consistent with the fact that older adults are less likely to use primary indicators to make trust judgments (Bailey and Leon, 2019). On the other hand, processing older faces requires more attentional resources. N170 and VPP components have been found to be involved in the automatic perception and construction of face structures. Although previous studies have suggested that the VPP and N170 components represent two opposite components from the same source (Ebner et al., 2010; Tortosa et al., 2013), some studies have suggested that the VPP component represents more complex face encoding than the N170 component (Joyce and Rossion, 2005). Unlike the N170 component only representing bottom-up signals, the VPP component was also shown to be modulated by top-down signals (Lu et al., 2017). Future studies are encouraged to further analyze the differences between these two components in trust decisions based on facial appearances.

The results of the FRN further indicated that the participants in the two groups may have differed in the evaluation of social attributes for younger and older faces. On the one hand, previous studies have analyzed the FRN component in the feedback stage of economic tasks (Gehring and Willoughby, 2002; Chen et al., 2012; Leng et al., 2020a). For example, in the ultimatum game, participants served as the responders and needed to accept or reject the fair or unfair allocation plan presented by the proposers. In general, the participants received the allocation after seeing the face of proposer. However, Leng et al. (2020b) presented the face of proposer after presenting the fair or unfair allocation plan to the responder, who was played by the participants. They found that compared with the fair allocation plan given by the proposer with a trustworthy face, the unfair allocation plan elicited more negative FRN. At the same time, when presenting the unfair allocation plan, trustworthy faces elicited more negative FRN than untrustworthy faces. On the other hand, some studies have also analyzed the FRN component in the face presentation stage. For example, after reinforcement learning, when the participants saw a face whose previous feedback amount was less than the amount provided, a more negative FRN was activated (Li et al., 2017). The results of this study showed that younger faces elicited a more negative FRN component than older faces in younger adults, whereas there was no significant difference in the activation of the FRN component based on the two kinds of faces in older adults. Furthermore, a more negative FRN component was activated in younger adults than in older adults for both younger and older faces. This result was consistent with the behavioral results that younger adults evaluated the social attributes of older faces more positively and then made the decision to invest more money for older faces. Older adults evaluated the social attributes of the two kinds of faces with no significant difference, which was also reflected in their behavioral decisions. Previous studies have pointed out that the FRN component can be used as an indicator of social evaluation (Pegna et al., 2019). Therefore, when analyzing the components in the face presentation stage, the differences in the FRN component in trustor and trustee age may have also led to differences in subsequent behavioral decisions.

Finally, the LPP results showed that individuals mobilized attentional resources in a top-down way during late face processing. Studies on LPP amplitudes have consistently found that it increases with motivation (Schupp et al., 2010; Chen et al., 2012). In one of the few ERP studies exploring facial trust, Yang et al. (2011) compared the relationship between LPP amplitudes and face trustworthiness ratings and found that untrustworthy faces elicited larger LPP amplitudes. It is likely that individuals allocate more attention to untrustworthy faces and with increasing LPP amplitudes. The results of this study showed that older faces elicited larger LPP amplitudes than younger faces in older adults. The effect of facial age on LPP amplitude indicated that individuals process faces not only in a bottom-up way, which elicits differences in the early face processing stage, but also in a top-down way in the late stage of face presentation. These findings are consistent with Marzi et al. (2014), who found that the evaluation of trustworthiness was a result not only of top-down influences, such as cognitive strategies and expectations, but also of bottom-up influences. Overall, it is possible that older adults make trust judgments that are less dependent on facial age because of the extensive experiences from their lifetimes (Bailey and Leon, 2019). The results of the N170 and LPP components showed that based on extensive experience, older adults also extracted cues of facial age in the late face processing stages.

In conclusion, both younger and older adults can extract and process facial age cues in early face processing, and younger adults then make trust judgments based on facial age in an automatic way. However, in the later stages of face processing, based on their personal experiences, older adults attend more to older faces and further process the cues of facial age, then making the trust judgments without the impact of facial age. Therefore, there is a difference in investments between younger and older faces for younger adults due to their greater reliance on facial age when making trustworthiness judgments in an automatic way, but we failed to find this difference for older adults due to mediation by top-down and bottom-up processes. From the N170 and VPP analyses, older adults were more likely to extract facial age cues automatically, similar to younger adults; they again extracted facial age in the late face processing stages to make trust judgments without relying on facial age.

## Conclusion

In this study, we used the trust game task to explore whether there were differences in facial trust judgments between groups of younger and older adults and further analyzed whether such differences had already existed in the face processing stage. It was found that only younger adults invested more in older faces than in younger faces. The ERP results showed that (1) in the early stage of face processing, younger faces elicited a larger N170 amplitude in the right hemisphere than older faces. At the same time, older faces elicited a larger VPP amplitude than younger faces, and younger adults had more positive VPP than older adults, which indicates that the facial age factor was indeed extracted in the early stage of face processing. (2) Regarding the social evaluation of faces, younger adults had larger FRN amplitudes when seeing younger faces than when seeing older faces, whereas there was no difference between the two kinds of faces for older adults, which indicated that younger adults evaluated the social attributes of older faces more positively. (3) In the later stage of face processing, only older adults had larger LPP activations when seeing older faces compared to the younger faces, which suggests that older adults further extracted the facial age factor in the later stage of face processing. Above all, we found a dissociation between the process of facial age cues and trust behaviors in both younger and older adults, which may provide insight into how to prevent older adults from being deceived.

## Data Availability Statement

The original contributions presented in the study are included in the article/supplementary material, further inquiries can be directed to the corresponding author/s.

## Ethics Statement

The studies involving human participants were reviewed and approved by the Institute of Psychology, Chinese Academy of Sciences. The patients/participants provided their written informed consent to participate in this study. Written informed consent was obtained from the individual(s) for the publication of any potentially identifiable images or data included in this article.

## Author Contributions

Z-WC and K-XW performed the experiments and wrote the manuscript. Y-NL and YQ designed the experiments, analyzed the data, and revised this manuscript. XL revised the manuscript. All authors contributed to the article and approved the submitted version.

## Conflict of Interest

The authors declare that the research was conducted in the absence of any commercial or financial relationships that could be construed as a potential conflict of interest.

## Publisher’s Note

All claims expressed in this article are solely those of the authors and do not necessarily represent those of their affiliated organizations, or those of the publisher, the editors and the reviewers. Any product that may be evaluated in this article, or claim that may be made by its manufacturer, is not guaranteed or endorsed by the publisher.
